# Relapse of Palmoplantar Pustulosis Following COVID-19 Vaccination

**DOI:** 10.7759/cureus.28604

**Published:** 2022-08-30

**Authors:** Shiho Katsuie, Kenta Nakamura, Eisaku Ogawa, Fuyuko Arakura, Ryuhei Okuyama

**Affiliations:** 1 Department of Dermatology, Shinshu University School of Medicine, Matsumoto, JPN; 2 Division of Dermatology, National Hospital Organization Matsumoto Medical Center, Matsumoto, JPN

**Keywords:** remission, relapse, pustular psoriasis, palmoplantar pustulosis, covid-19 vaccination

## Abstract

Palmoplantar pustulosis (PPP) is a rare chronic pustular condition that affects the palms and soles. Smoking and focal infections and dental metal allergies are risk factors for PPP development. Here we report a case of a 60-year-old woman who experienced a relapse of PPP after receiving the COMIRNATY vaccine against COVID-19. The patient relapsed after being in remission for seven years. This article shows the possible implications of COVID-19 vaccination related to the relapse of previous diseases and stresses the importance of careful observation of post-vaccination occurrences of skin eruptions, especially in patients having a history of PPP.

## Introduction

Palmoplantar pustulosis (PPP) is a chronic disease characterised by sterile pustules that predominantly occur on the palms and soles [1]. PPP is closely associated with smoking, focal infections and dental metal allergies [2]. Cutaneous adverse effects following coronavirus disease-19 (COVID-19) vaccination were described, including local injection-site reactions, urticarial eruptions, morbilliform reactions and pustular psoriasis [3-5]. In Europe and North America, PPP is thought to be a localized type of pustular psoriasis. However, there are no reports of PPP after COVID-19 vaccination. Here, we report a case of PPP that was in remission for seven years and relapsed after vaccination with COMIRNATY, which is an mRNA vaccine manufactured by Pfizer against COVID-19.

## Case presentation

**Figure 1:**
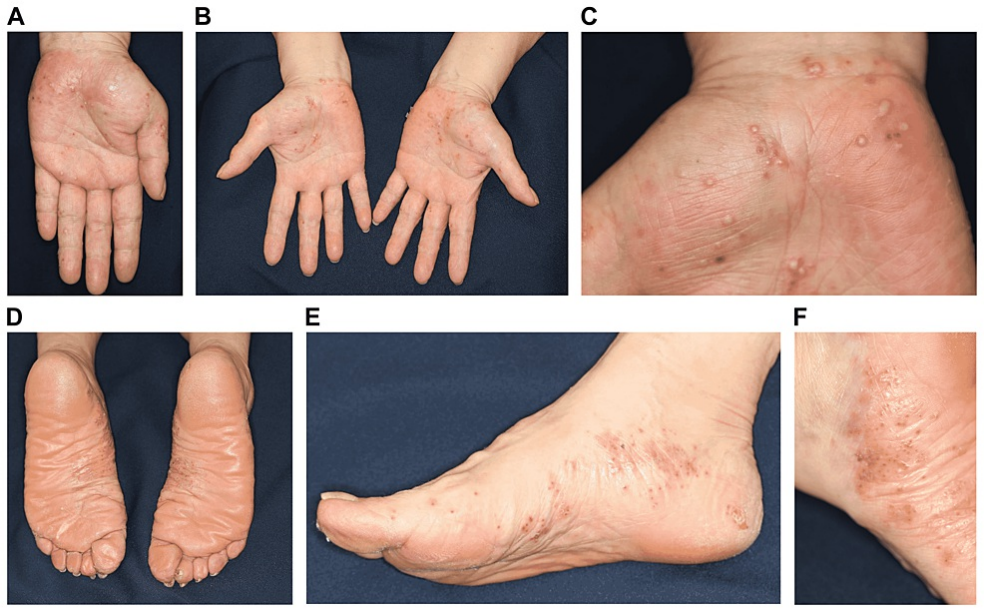
Clinical images showing small pustules sporadically on the palms and soles (a)-(f).

A 60-year-old Japanese woman who had PPP for 25 years was being treated with topical steroids (Difluprednate Ointment 0.05%) at a dermatology clinic. She had no past medical history and was on no other medications. Her rash showed repeated improvement and aggravation. She was allergic to metals (palladium and nickel) and had a history of smoking (20 cigarettes per day for 40 years). We advised her about smoking cessation, but she was not able to discontinue smoking. Eight years ago, the patient was enrolled in a clinical trial (phase II) for an anti-interleukin (IL)-23p19 monoclonal antibody formulation in our hospital (Figure 1a). On administering the investigational agent twice, the palmoplantar rash completely disappeared, and PPP remained in remission. One week after being administered with the first dose of COMIRNATY, pustules began appearing on the palms and soles. On receiving the second dose, the patient presented to our department with a rash that had gradually worsened. On arrival, multiple pustules were observed on the palms and soles (Figures 1b-1f). These pustules are thought to be sterile because bacterial cultures obtained from the pustules are negative. There was no eruption on any other part of the skin or on the oral mucous membrane. Although the patient experienced pain at the injection site upon vaccination, fever was absent. Furthermore, she reported no recent viral illness. She was also not exposed to stress, palladium, or nickel. Because PPP relapsed immediately after the vaccination, a diagnosis of PPP recurrence caused by vaccination was made. The rash partially improved by topical steroid application for two weeks. Thus far, there are no reports of the relapse of the eruption.

## Discussion

While PPP is often categorised as an acral variant of localised pustular psoriasis, it is an entity distinct from pustular psoriasis [1]. Recently, relapse and aggravation of psoriasis following COVID-19 vaccinations were reported [3]. Furthermore, generalised pustular psoriasis occurred de novo after the vaccinations [4,5]. However, to the best of our knowledge, no studies of PPP recurrence immediately after COVID-19 vaccinations have been reported. Previously, there have been reports of newly developed psoriasis and aggravated psoriasis cases following influenza, tetanus-diphtheria, BCG, and pneumococcal pneumonia vaccinations [6-13]. It is inferred that flu vaccines may exacerbate psoriasis by inducing Th1 and Th17 immune responses [6]. In addition, elevated Th17 responses have been observed in patients with severe COVID-19 infection [14]. In PPP, Th17 contributes to neutrophilic activation and infiltration through the IL-23/IL-17 pathway [15], and vaccinations may activate Th17 immune responses. In 2018, guselkumab of the anti-IL23p19 antibody became the insurance application for intractable PPP in Japan. The elucidation of the mechanism of the PPP is expected by analysis of this treatment.

## Conclusions

To summarise, we encountered a case of PPP that relapsed following COVID-19 vaccination after seven years of remission. This is the first report of PPP recurrence following COVID-19 vaccination. The mechanism by which PPP was caused was unknown, but Th17 might be involved. Additional work is needed to confirm this. Our case demonstrates the necessity to carefully observe the recurrence and possible exacerbation of PPP after COVID-19 vaccination. Of course, the appearance of such side effects does not preclude taking the vaccine in the future.

## References

[1] Yamamoto T (2021). Similarity and difference between palmoplantar pustulosis and pustular psoriasis. J Dermatol.

[2] Misiak-Galazka M, Zozula J, Rudnicka L (2020). Palmoplantar pustulosis: recent advances in etiopathogenesis and emerging treatments. Am J Clin Dermatol.

[3] McMahon DE, Amerson E, Rosenbach M (2021). Cutaneous reactions reported after Moderna and Pfizer COVID-19 vaccination: a registry-based study of 414 cases. J Am Acad Dermatol.

[4] Elamin S, Hinds F, Tolland J (2022). A case of de novo generalised pustular psoriasis following Oxford-Astra Zeneca COVID-19 vaccine. Clin Exp Dermatol.

[5] Onsun N, Kaya G, Işık BG, Güneş B (2021). A generalized pustular psoriasis flare after CoronaVac COVID-19 vaccination: case report. Health Promot Perspect.

[6] Gunes AT, Fetil E, Akarsu S, Ozbagcivan O, Babayeva L (2015). Possible triggering effect of influenza vaccination on psoriasis. J Immunol Res.

[7] Raaschou-Nielsen W (1955). Psoriasis vaccinalis; report of two cases, one following B.C.G. vaccination and one following vaccination against influenza. Acta Derm Venereol.

[8] Sbidian E, Eftekahri P, Viguier M (2014). National survey of psoriasis flares after 2009 monovalent H1N1/seasonal vaccines. Dermatology.

[9] Shin MS, Kim SJ, Kim SH, Kwak YG, Park HJ (2013). New onset guttate psoriasis following pandemic H1N1 influenza vaccination. Ann Dermatol.

[10] Shi CR, Nambudiri VE (2017). Widespread psoriasis flare following influenza vaccination. Vaccine.

[11] Macias VC, Cunha D (2013). Psoriasis triggered by tetanus-diphtheria vaccination. Cutan Ocul Toxicol.

[12] Yoneyama S, Kamiya K, Kishimoto M, Komine M, Ohtsuki M (2019). Generalized exacerbation of psoriasis vulgaris induced by pneumococcal polysaccharide vaccine. J Dermatol.

[13] Grafanaki K, Vryzaki E, Georgiou S, Liga M (2020). Double trouble: influenza and pneumococcal vaccine exacerbation of psoriasis in a new-onset polycythemia vera patient. J Dermatol.

[14] Wu D, Yang XO (2020). TH17 responses in cytokine storm of COVID- 19: an emerging target of JAK2 inhibitor Fedratinib. J Microbiol Immunol Infect.

[15] Torii K, Furuhashi T, Saito C (2011). Increased peripheral Th17 in patients with pustulosis palmaris et plantaris. Arch Dermatol Res.

